# Impact of electromagnetic fields on stem cells: common mechanisms at the crossroad between adult neurogenesis and osteogenesis

**DOI:** 10.3389/fncel.2015.00228

**Published:** 2015-06-15

**Authors:** Lucia Leone, Maria Vittoria Podda, Claudio Grassi

**Affiliations:** Institute of Human Physiology, Medical School, Università Cattolica del Sacro CuoreRome, Italy

**Keywords:** epigenetics, extremely-low frequency electromagnetic fields, gene expression programs, mesenchymal stem cells, neural stem cells

## Abstract

In the recent years adult neural and mesenchymal stem cells have been intensively investigated as effective resources for repair therapies. *In vivo* and *in vitro* studies have provided insights on the molecular mechanisms underlying the neurogenic and osteogenic processes in adulthood. This knowledge appears fundamental for the development of targeted strategies to manipulate stem cells. Here we review recent literature dealing with the effects of electromagnetic fields on stem cell biology that lends support to their use as a promising tool to positively influence the different steps of neurogenic and osteogenic processes. We will focus on recent studies revealing that extremely-low frequency electromagnetic fields enhance adult hippocampal neurogenesis by inducing epigenetic modifications on the regulatory sequences of genes responsible for neural stem cell proliferation and neuronal differentiation. In light of the emerging critical role played by chromatin modifications in maintaining the stemness as well as in regulating stem cell differentiation, we will also attempt to exploit epigenetic changes that can represent common targets for electromagnetic field effects on neurogenic and osteogenic processes.

## Introduction

Any adult tissue with repair/regenerative capabilities contains tissue-specific stem cells (SCs) defined as clonogenic, self-renewing cells that retain proliferative and differentiation potential allowing to preserve tissue homeostasis and to repair injury (Anderson et al., 2001). Unlike differentiated cells, adult SCs are unspecialized cells that can self-renew to replenish themselves and differentiate into one or more specialized cell types within a committed lineage (Minguell et al., 2001). As such SCs hold promise for tissue/organ repair with the ultimate goal to regenerate and restore normal functions. Adult SCs are most often in a quiescent state, and either or both intrinsic or extrinsic factors can trigger programs for self-renewal or differentiation (Kobilo et al., 2011; Podda et al., 2013; Leone et al., 2014). It is currently accepted that a combination of niche signals and cell intrinsic programs orchestrate the transition from an undifferentiated stem cell state to a progenitor cell committed to the final fate. Among multiple sources of adult stem cells, neural SCs (NSCs) and mesenchymal SCs (MSCs) have been intensively studied for their role in brain and bone physiology as well as for their potential use in cell-based therapies for treating neurological/neurodegenerative diseases and for reconstructive surgery, respectively (Yamaguchi, 2014; Hayrapetyan et al., 2015; Lin and Iacovitti, 2015).

In this context, great effort has been put to identify stimuli and molecular pathways influencing the neurogenic and osteogenic processes. Within this scenario here we review recent literature focusing on epigenetic mechanisms that appear to be crucially involved in the process of both neurogenesis and osteogenesis. We will also discuss the involvement of chromatin modifications in mediating the effects of extremely-low frequency electromagnetic field (ELFEF) stimulation that is emerging as an effective tool to positively modulate neurogenic and osteogenic processes.

## Neural Stem Cells

In the adult mammalian brain, NSCs reside mainly in two discrete regions: the subgranular zone of the hippocampal dentate gyrus and the subventricular zone of the lateral ventricles (Gage, 2000; Ming and Song, 2011). Throughout life these neurogenic niches ensure continuous production of new neurons and maintain the NSC pool (Kempermann et al., 2004). NSC self-renewal is intrinsically sustained by specific “stemness” genes, including those controlled by Notch signaling (Louvi and Artavanis-Tsakonas, 2006; Ables et al., 2010). NSC differentiation results from the gradual inactivation of “stemness” genes and the activation of pro-neural genes including, *Ascl1* (Achaete-Scute Complex-Like 1, also known as *Mash1*), *Neurogenin1* and *NeuroD1*.

Recent studies have also revealed a key role of Wnt/β-catenin signaling in balancing NSC self-renewal and neuronal differentiation. In particular, NeuroD1 has been reported to be the downstream mediator of Wnt pathway and its expression is silenced in undifferentiated NSCs. In the presence of extracellular Wnt, β-catenin accumulates in the nucleus, resulting in NeuroD1 activation and subsequent neuronal differentiation (Kuwabara et al., 2009). A similar molecular mechanism has been described for the transcription factor cAMP response element-binding protein (CREB), which modulates neuronal differentiation by binding regulatory sequences of pro-neural genes (Deisseroth et al., 2004; Jagasia et al., 2009). In particular, Ca^2+^ signaling triggers phosphorylation of CREB, that, once activated, promotes NSC differentiation (West et al., 2001; Giachino et al., 2005; D’Ascenzo et al., 2006; Leone et al., 2014).

## Mesenchymal Stem Cells (MSCs)

MSCs are generally derived from the bone marrow (Friedenstein et al., 1987; Pittenger et al., 1999; Lin et al., 2013), but they can also be sourced from other tissues including umbilical cord blood and adipose tissue. MSCs give rise to mesenchymal phenotypes including bone, cartilage and fat, and to non-mesenchymal cells including neural cells. Numerous studies, primarily focusing on bone cell lineages, have been performed to get insight into MSC differentiation process (Minguell et al., 2001; Fakhry et al., 2013).

Bone formation is regulated by osteogenic transcription factors that mediate the staged expression of bone phenotypic genes, such as the osteocalcin (OC) gene, during differentiation of osteoprogenitor cells to mature osteoblasts. In particular, signaling molecules such as bone morphogenetic proteins (BMPs) and Wnt pathway favor osteoblastogenesis, while Notch1 and its downstream target Hes1 inhibit osteoblast differentiation. Recently, it has been shown that the transcriptional factor Runx2, a major target of BMP pathway, induces osteoblast differentiation by repressing *Hes1* and by activating *OC* and other bone-related genes (Ann et al., 2011; Wang et al., 2013).

## Epigenetic Mechanisms in Neurogenesis and Osteogenesis

Increasing body of evidence supports the view that epigenetic mechanisms including DNA methylation and histone modifications orchestrate SC self-renewal, lineage commitment, cell fate specification and terminal differentiation. These regulatory mechanisms promote the formation of relatively “open” and “poised” epigenetic states that, by regulating transcriptional activity, mediate the execution of lineage-specific gene expression programs.

Consistent with this concept, transcriptional control of both adult neurogenesis and osteogenesis is under intensive regulation by epigenetic modifications of the regulatory sequences of pro-neural genes including *Ascl1*, *Neurogenin1* and *NeuroD1* (Ma et al., 2010; Hsieh, 2012; Eslaminejad et al., 2013; Amador-Arjona et al., 2015) and bone-related genes such as *OC* (Gutierrez et al., 2002; Eslaminejad et al., 2013), respectively.

### DNA Methylation

DNA methylation refers to addition of methyl group to the carbon 5 position of the DNA base cysteine, which results in the generation of 5-methylcytosine (5-mC). DNA methylation is catalyzed by DNA-methyl-transferase (DNMT) and usually results in gene repression. DNMT3a and DNMT3b establish *de novo* methylation, whereas DNMT1 maintains methylation patterns during cell division. *De novo* methylation and maintenance of methylation marks, either directly or indirectly affecting gene expression, are capable of regulating sequential steps of adult neurogenesis (Covic et al., 2010; Hsieh and Eisch, 2010).

Seemingly, DNA methylation is dynamically involved in MSC bone differentiation. A significant hypermethylation at the *OC* locus has been associated with its repression. Accordingly, during osteoblast differentiation this CpG methylation significantly decreased, resulting in enhanced *OC* expression (Villagra et al., 2006). Furthermore, Dansranjavin et al. (2009) demonstrated that MSC differentiation into osteoblast cells was accompanied by reduced expression of the stemness genes via hypermethylation of their promoters.

### Histone Modifications

Gene expression also depends on DNA accessibility, which is determined by histone post-transcriptional modifications, such as acetylation and methylation that commonly activate and repress gene expression, respectively. These modifications have been involved in both adult neurogenesis and osteogenesis (Hsieh and Eisch, 2010; Ma et al., 2010). Histone acetylation is a dynamic process regulated by both histone acetyltransferases (HATs) and deacetylases (HDACs) that add or remove acetylation marks, respectively. Transcriptional repression through HDAC activity is essential for adult NSC proliferation and self-renewal. For example, the expression of the Notch effector, Hes1, regulates NSC self-renewal by interacting with different HDACs to repress pro-neural gene expression (Hsieh et al., 2004; Kuwabara et al., 2009; Sun et al., 2011; Zhou et al., 2011). On the other hand, enhanced adult NSC differentiation has been associated with increased H3 acetylation levels and the expression of CREB-binding protein (CBP), a critical histone acetyltransferase (HAT) for neuronal differentiation (Chatterjee et al., 2013). Thus, maintenance of histone acetylation appears important for neuronal lineage progression of adult NSCs, while histone deacetylation seems relevant for NSC self-renewal.

Histone acetylation/deacetylation has also been involved in osteogenesis. Acetylation of histone H4 and to a lesser extent, of H3 at the *OC* promoter accompanies the induction of *OC* expression in mature osteoblasts (Shen et al., 2003). Accordingly, the down-regulation of HDAC1 is associated to osteoblast differentiation (Lee et al., 2006).

Adult neurogenesis and osteogenesis are also under tight epigenetic control of histone methylation that is regulated by two antagonistic complexes: (i) Polycomb (PcG), that promotes H3 lysine 27 tri-methylation (H3K27me3); and (ii) Trithorax (TrxG), that promotes H3 lysine 4 tri-methylation (H3K4me3).

In NSCs, depletion of PcG components, such as Ezh2, largely removed H3K27me3 markers, de-repressed a wide panel of genes, and delicately altered the balance between self-renewal and differentiation as well as the timing of neurogenesis (Hsieh and Eisch, 2010; Pereira et al., 2010).

Osteogenic lineage determination has been associated to chromatin hyperacetylation and H3K4 hypermethylation of different genes, including *OC* (Hassan et al., 2007; Wei et al., 2011).

**Figure 1:**
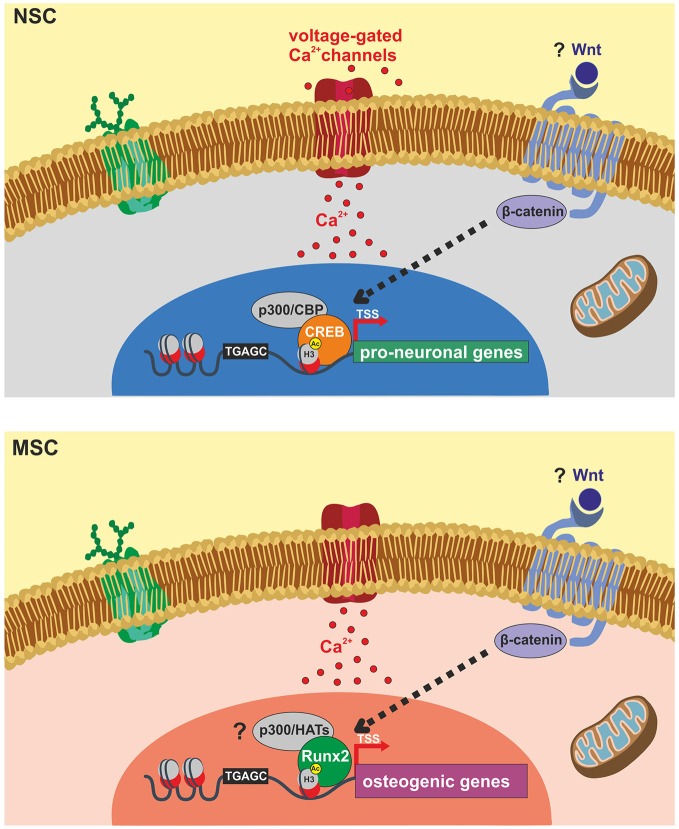
**Schematic representation of identified molecular targets involved in ELFEF-induced enhancement of adult hippocampal neurogenesis and osteogenesis**. Experimental evidence demonstrates that ELFEFs affect key molecular players involved in the up-regulation of pro-neuronal genes in hippocampal neural stem cells (NSCs, upper panel) and bone-related genes in mesenchymal stem cells (MSCs, lower panel). In hippocampal NSCs ELFEFs enhance pro-neuronal gene expression by a mechanism involving: (i) Ca^2+^-dependent phosphorylation/activation of CREB and its binding on pro-neuronal gene promoters; (ii) increased recruitment of the HAT CREB-binding protein (CBP) on the same regulatory sequences; (iii) enhanced histone 3 (H3) acetylation on lysine 9 (H3Ac) of pro-neuronal gene promoters (i.e., *NeuroD1* and *Neurogenin1*). In MSCs ELFEF-induced up-regulation of bone-related genes has been also linked to intracellular Ca^2+^ signaling and enhanced expression of the transcription factor Runx2 which is known to bind the regulatory sequences of osteogenic genes promoting their expression. Question marks indicate putative common molecular targets of ELFEFs in NSCs and MSCs including: (i) Wnt/β-catenin signaling regulating pro-neuronal and osteogenic gene expression at transcriptional and epigenetic levels; (ii) activation of p300 or other HATs by Runx2, resembling the pCREB/CBP pathway activated by ELFEFs in hippocampal NSCs.

The literature reviewed above highlights a prominent role of epigenetic mechanisms in the modulation of gene expression during neurogenesis and osteogenesis processes. Interestingly, experimental evidence involved these mechanisms in the beneficial effects of ELFEF stimulation on adult hippocampal neurogenesis (Figure 1).

## Effects of Electromagnetic Fields on Neural and Mesenchymal Stem Cells

It is widely reported that electromagnetic fields modulate different steps of neurogenesis and osteogenesis and several potential cellular targets have been identified. However, the heterogeneity of exposure systems and experimental protocols chosen has produced a complex picture in which data may appear at first sight inconsistent. On the other hand, when comparing data obtained under similar exposure conditions then they appear more homogeneous (Di Lazzaro et al., 2013). From this perspective here we focused on ELFEFs and documented that such stimulation effectively promotes proliferation and functional differentiation of both NSCs and MSCs, likely engaging similar molecular pathways.

With regard to NSCs, our initial studies showed that ELFEF stimulation (1 mT, 50 Hz) enhanced differentiation of adult cortical NSCs (Piacentini et al., 2008). In line with what reported in other cell models (Grassi et al., 2004a; Wolf et al., 2005), ELFEF stimulation increased proliferation of undifferentiated NSCs but, once the differentiation process had started, ELFEFs inhibited proliferation and increased the percentage of cells acquiring molecular markers and functional properties of neurons. Molecular and electrophysiological data showed that these effects were linked to enhanced expression and function of voltage-gated L-type calcium channels (Ca_v_1) (Grassi et al., 2004b; D’Ascenzo et al., 2006; Piacentini et al., 2008). These findings prompted subsequent studies (Cuccurazzu et al., 2010) aimed at investigating the effects of ELFEFs on the expression of genes regulating NSC fate given the well-recognized prominent role played by intracellular Ca^2+^ signaling in such mechanisms (West et al., 2001; Deisseroth et al., 2004). In particular, i*n vivo* and *in vitro* studies on adult hippocampal neurogenesis demonstrated that ELFEF-induced Ca^2+^ influx through Ca_v_1 channels led to increased CREB phosphorylation and that was a crucial step in regulating the expression of genes responsible for NSC proliferation and neuronal differentiation (Cuccurazzu et al., 2010). Indeed, quantitative RT-PCR analysis of hippocampal extracts from adult mice exposed to ELFEFs (50 Hz, 1 mT; 7 h/day for 7 days) revealed increased transcription of *Ascl1*, *NeuroD2*, and *Hes1* paralleled by higher levels of mRNA encoding α_1C_ and α_1D_ subunits of Ca_v_1.2 and Ca_v_1.3 channels. Enhanced expression of NeuroD1, NeuroD2, and the Ca_v_1 channel proteins in the hippocampi of ELFEF-exposed mice was also confirmed by Western blot analysis. Immunofluorescence analyses revealed that *in vivo* ELFEF stimulation affected NSC proliferation and neuronal differentiation, as shown by increased numbers of cells labeled for the proliferation marker 5-bromo-2′-deoxyuridine (BrdU), and double-labeled for BrdU and the immature neuronal marker doublecortin. Interestingly, 30 days after the end of the ELFEF stimulation protocol ~50% of the newborn neurons became mature granule cells that were functionally integrated in the dentate gyrus network, as demonstrated by neurophysiological indexes. In particular, in hippocampal brain slices from ELFEF exposed mice, long-term potentiation at medial perforant path-dentate granule cell synapses in the presence and in the absence of GABA_A_ receptor blockade was significantly greater than that observed in unexposed control mice (Cuccurazzu et al., 2010), as expected as a consequence of enhanced number of newborn neurons integrated in the local circuit (Massa et al., 2011).

In a subsequent study we demonstrated that *in vivo* ELFEF stimulation also promoted the survival of hippocampal newly generated neuron by rescuing them from apoptotic cell death, an effect associated with enhanced expression of pro-survival protein Bcl-2 and decreased expression of the apoptotic protein Bax (Podda et al., 2014).

Importantly, our most recent study demonstrated that the ELFEF-induced enhancement of hippocampal neurogenesis and synaptic plasticity lead to improved hippocampal-dependent learning and memory in mice (Leone et al., 2014). This study shed further light on molecular mechanisms underlying ELFEFs’ effects revealing a significant regulation of epigenetic mechanisms leading to pro-neuronal gene expression. In particular, in *in vitro* and *in vivo* models of adult hippocampal neurogenesis we demonstrated that enhanced expression of *Hes1* in proliferating NSCs and *NeuroD1*, and *Neurogenin1* in differentiating NSCs were associated to increased H3K9 acetylation and Ca^2+^-dependent CREB/CBP recruitment on the regulatory sequence of these genes (Leone et al., 2014). This study suggested that regulation of epigenetic mechanism provides a fine and targeted control of neurogenic process by ELFEFs.

Concerning MSCs, it is worth noting that, although the neuronal transdifferentiation of somatic SCs for reparative strategies in neurodegenerative diseases is still debated (Lu et al., 2004), several studies reported the effects of 50 Hz ELFEFs in promoting neuronal differentiation of MSCs from various sources including bone marrow, supporting a strong effects of this stimulation on pro-neurogenic pathways.

The work by Cho et al. (2012) showed that ELFEFs (50 Hz, 1 mT for 12 days) increased neuronal differentiation of human bone marrow-derived (hBM)-MSCs, inducing the expression of neural cell markers including NeuroD1. Similar results were obtained by Bai et al. (2013) using similar ELFEF parameters (50 Hz, 5 mT for 12 days). More recently, Seong et al. (2014) showed that ELFEF exposure (50 Hz, 1 mT for 8 days) of hBM-MSCs promoted neuronal differentiation even in the absence of any neurotrophic factor. Indeed, exposed hBM–MSCs showed significant increase of NeuroD1 expression as well as electrophysiological properties of neurons. The same authors demonstrated that ELFEFs enhanced differentiation of mouse NSCs towards the neuronal phenotype. Analysis of the transcriptome of ELFEF-exposed hBM-MSCs and mouse NSCs revealed dramatic changes in global gene expression in both cell types compared to unexposed cells, with relevant up-regulation of several transcription factors, such as Egr1, DNA-binding protein inhibitor ID-1 and Hes1. In particular, Egr1, regarded as a strong early neurogenic transcription factor, appeared to be the most highly upregulated in neuronal differenting cultures from hBM-MSCs and mouse NSCs. Seong et al. (2014) further confirmed the role of Egr1 in mediating the pro-neurogenic effect of ELFEFs on MSCs showing that: (i) knockdown of Egr1 in the hBM–MSCs significantly inhibited ELFEF induced neuronal differentiation; (ii) the overexpression of Egr1 combined with ELFEF exposure increased the efficiency of cell-replacement therapy thus alleviating neurological symptoms in a Parkinson’s disease mouse model.

Besides the key finding of the study involving the transcription factor Egr1 in ELFEF effects, it is interesting to note that the list of genes modulated by ELFEFs includes HDACs (i.e., HDAC5 and HDAC11) that are known to critically regulate SC self-renewal and differentiation (Cheng et al., 2005; Sun et al., 2011; Zhou et al., 2011). Unfortunately, the study by Seong and co-workers did not specifically address the issue of whether histone modifications were involved in ELFEF mediated up-regulation of neuronal genes.

Besides the studies exploring the potential to promote neuronal transdifferentiation of MSCs, ELFEFs have been well known for many years as potent stimuli to promote ostegenesis and cartilage formation (Heckman et al., 1981). In this respect the majority of studies were performed by using pulsed EFs (PEMF, frequencies in the range of 7.5–75 Hz) and, given their efficacy, devices producing such stimuli are currently approved by the US Food and Drug Administration for the treatment of fracture non-unions and osseous defects (Assiotis et al., 2012; Boyette and Herrera-Soto, 2012). Initially, clinical effectiveness of EFs was attributed to the accelerated formation of bone matrix by the weak electric current generated by the magnetic field (de Haas et al., 1980; Aaron and Ciombor, 1996). However, more recent studies have clearly involved MSCs as target of EF action.

Indeed, studies performed on bone marrow-derived stromal cells (BMSCs) demonstrated that exposure to PEMF stimulates cell proliferation as well as osteogenesis by increasing early osteogenic markers including Runx2/Cbfa1 and alkaline phosphatase (ALP; Pittenger et al., 1999; Sun et al., 2009; Tsai et al., 2009).

The effects of PEMFs on osteogenic differentiation of adipose-derived stem cells (ASCs) have been more recently investigated. In particular, PEMF treatment enhanced the expression of bone matrix genes (OC and collagen type I in ASC) as well as bone mineralization (Ceccarelli et al., 2013; Chen et al., 2013; Ongaro et al., 2014). Additionally, recent lines of evidence suggest that sinusoidal ELFEF stimulation promotes proliferation and osteogenic differentiation of both BMSCs (Zhong et al., 2012) and ASCs (Kang et al., 2013).

At present the mechanism by which PEMFs/ELFEFs promote the formation of bone remains elusive and future studies are highly demanded. Some evidences indicate that, as documented for NSCs, the electromagnetic stimulation raises the net Ca^2+^ flux and expression/activation of Ca^2+^-binding proteins such as calmodulin in human osteoblast-like cells and MSCs (Fitzsimmons et al., 1994; Lim et al., 2013). The increase in the cytosolic Ca^2+^ concentration is the starting point for signaling pathways targeting specific bone matrix genes and, in keeping with this, the application of the electromagnetic waves was shown to increase the level of transcripts of osteogenesis-related genes including those encoding for decorin, osteopontin, collagen type-I and Runx2 (Figure 1).

## Conclusions

The recent findings in stem cell biology have opened a new window in the expanding area of regenerative medicine based on tissue engineering and cell therapy derived from a variety of SCs, including NSCs and MSCs.

With regard to neurogenesis and ostegenesis it is becoming increasingly clear that these processes rely on the activation of specific and complex transcriptional programs whose regulation may provide a cellular candidate for therapeutic intervention. In this context epigenetic mechanisms play a critical regulatory role translating a wide array of endogenous and exogenous signals into persistent changes in gene expression in both NSCs and MSCs. ELFEF stimulation has been recognized as effective tool in promoting both neurogenesis and osteogenesis and studies performed so far on NSCs point to chromatin remodeling as a critical determinant in ELFEF’s induced pro-neuronal gene expression. The literature here reviewed suggests that epigenetic regulation of bone-related gene may seemingly mediate the effects exerted by EFs on osteogenesis.

It is our opinion that future research on different types of SCs may benefit from higher degree of communication between the different fields that would contribute to uncover more than expected common molecular pathways and stimulation paradigms of potential relevance for therapeutic interventions.

## Conflict of Interest Statement

The authors declare that the research was conducted in the absence of any commercial or financial relationships that could be construed as a potential conflict of interest.
